# The global challenge of antimalarial drug resistance

**DOI:** 10.1186/1475-2875-11-S1-O37

**Published:** 2012-10-15

**Authors:** Pascal Ringwald, Amy Barrette, Charlotte Rasmussen, Robert D Newman

**Affiliations:** 1 WHO Global Malaria Programme (GMP), Geneva, Switzerland

## 

The continued geographic expansion of *Plasmodium falciparum* resistance to artemisinin derivatives is an urgent public health concern. The last decade has witnessed dramatic progress in global malaria control and elimination efforts, in large part because of the scale-up of vector control interventions and improved access to effective antimalarials, especially artemisinin-based combination therapies (ACTs). Malaria cases have fallen by >50% in 43 countries since 2001. Since 2000, malaria mortality rates have declined by >25% globally, and by >33% in the WHO African Region. Malaria patients worldwide are dependent on the efficacy of ACTs. In 2010, more than 180 million ACT treatment courses were procured for the public sector alone.

Resistance to artemisinins was first confirmed on the Cambodia-Thailand border in late 2006, and reported in 2008. The first containment project - the Artemisinin Resistance Containment and Elimination (ARCE) project -was funded by BMGF and coordinated by WHO-GMP; it ran from late2008-2011. The project did not eliminate malaria from the affected area; however, active case detection, strengthened community case management and better vector control did result in a sustained and dramatic reduction in *Plasmodium falciparum* burden. To mobilize global and local stakeholders for containment and ultimately elimination of artemisinin resistance, WHO, together with RBM, launched the *Global Plan for Artemisinin Resistance Containment* (*GPARC*) in January 2011. The GPARC defines priorities to contain or eliminate artemisinin resistance where it already exists, or to prevent it where it has not yet appeared. While strong country-level activities are the central building blocks in the response, artemisinin resistance does not respect national boundaries. Resistance has generally been identified in areas with high numbers of migrants and close to national borders. Consequently, national responses to the threat of resistance are not sufficient; strong regional coordination is crucial.

Despite the emergence of artemisinin resistance in the Greater Mekong subregion, ACTs remain the most effective treatment for uncomplicated falciparum malaria; most patients with delayed response are cured if the partner drug remains effective. However, mefloquine resistance is widespread in Thailand and Cambodia, and piperaquine resistance may have emerged in Western Cambodia, limiting the number of treatment options for uncomplicated malaria. In addition to the existing foci, there is growing evidence of resistance to artemisinin, as defined by delayed parasite clearance times in south-eastern Myanmar and southern and central Vietnam. It is not known if these new foci represent spread or de novo emergence of artemisinin resistance.

In response to new data, containment efforts have been started in western Thailand, south-eastern Myanmar and Vietnam. These new programmes will draw on the lessons learned during the containment project in Cambodia and Thailand. In addition, routine monitoring must be strengthened for the early detection of artemisinin resistance, ensure that recommended first-line ACTs are effective, and that timely changes in treatment policies can be made. Many aspects of artemisinin resistance are still not well understood. Consequently, there is an urgent need for further research to refine our knowledge of artemisinin resistance, including the identification of molecular markers and better in vitro sensitivity tests. These efforts require full funding to ensure that artemisinin resistance does not spread and compromise global success in malaria control and elimination.

